# Higher HEI-2015 scores are associated with lower risk of gout and hyperuricemia: Results from the national health and nutrition examination survey 2007–2016

**DOI:** 10.3389/fnut.2022.921550

**Published:** 2022-08-03

**Authors:** Jiaqi Nie, Ming-Gang Deng, Kai Wang, Fang Liu, Haoling Xu, Qianqian Feng, Xiaosong Li, Yichi Yang, Ruyi Zhang, Suqing Wang

**Affiliations:** ^1^School of Public Health, Wuhan University, Wuhan, China; ^2^School of Nursing, Wuhan University, Wuhan, China; ^3^Center for Chronic Disease Rehabilitation, School of Nursing, Wuhan University, Wuhan, China

**Keywords:** healthy eating index, gout, hyperuricemia, daily diet, weighted quantile sum (WQS) regression, NHANES

## Abstract

Gout, the most prevalent inflammatory arthritis, is becoming increasingly prevalent in the United States and across the world, and it adversely impacts people’s quality of life and their health. Few studies have focused on the relationship between daily dietary quality and gout, so the topic requires further exploration. Data were derived from the National Health and Nutrition Examination Survey 2007–2016, and the inclusion criteria of the analytic sample were (1) adults, age ≥20 years, with complete information about HEI-2015, gout, and uric acid; (2) complete information of demographics, lifestyle (BMI, smoking, drinking), and disease history [hypertension, chronic kidney disease (CKD), diabetes]. The quality of the daily diet was reflected using the Healthy Eating Index 2015 (HEI-2015). The baseline features of different groups were examined using the Scott-Rao chi-square tests, and the association between the HEI-2015 score and the risk of gout/hyperuricemia (HUA) was investigated using weighted logistic regression models. The effects of different dietary components in the HEI-2015 on reducing the risk of gout/HUA were evaluated by weighted quantile sum (WQS) regression models. After adjusting for demographic characteristics, behavioral covariates, and disease history, higher HEI-2015 scores were associated with a significantly lower risk of gout (OR: 0.878, 95% CI: 0.876–0.880) and HUA (OR: 0.978, 95% CI: 0.976–0.979) in weighted logistic regression. Dairy, whole grains, plant proteins, and added sugar contributed greatly in HEI-2015 to reducing gout risk (weights of WQS index: 42, 17.18, 16.13, and 7.93%, respectively). Dairy, total fruits, greens and beans, and plant proteins contributed greatly in HEI-2015 to reducing HUA risk (weights of WQS index: 28.9, 17.13, 16.84, and 11.39%, respectively). As the result, adherence to the American Dietary Guidelines may assist to decrease the risk of gout/HUA in American adults, and greater emphasis should be placed on dairy products, whole grains, fruits, legumes, and added sugars.

## Introduction

Gout is inflammatory arthritis due to hyperuricemia (HUA), the most important pathological feature of which is the presence of sodium urate crystals in the joints. Gout episodes are frequently accompanied by excruciating joint pain and are closely linked to long-term conditions including obesity and hypertension (1–3), which impair the quality of life (4) and raise medical and care expenditures (5). There were 41.2 million prevalent cases of gout globally, with 7.4 million incident cases per year and almost 1.3 million years lived with disability (YLD) due to gout in 2017 (6, 7). Gout has become a global public health problem.

Recent research has revealed a strong link between diet and gout. According to a 26-year prospective cohort trial in the Health Professionals Follow-up Study (HPFS), the Dietary Approaches to Stop Hypertension (DASH) diet was beneficial in decreasing the incidence of gout and lowering serum uric acid concentrations in adult males, in comparison with a Western diet (8). Additionally, a clinical trial in Israel found that adherence to a Mediterranean diet can significantly lower serum uric acid and reduce the risk of gout in severely obese patients (9). Numerous studies have also evaluated the connection between particular foods or nutrients and gout, including dairy products (10), whole grains, fruits (8, 11, 12), added sugars (13, 14), and vitamins (12, 13). For instance, dairy products were shown to be beneficial in lowering serum uric acid and reducing the frequency of gout episodes in a randomized controlled study of gout patients (15). Moreover, a prospective cohort study showed that vegetables and fruits exhibited similar impacts to dairy products (11), and a cross-sectional analysis of the Brazilian Longitudinal Study of Adult Health (ELSA-Brazil) revealed that increased consumption of soft drinks and fructose was positively correlated with the risk of HUA (14).

The Healthy Eating Index (HEI) is a dietary quality indicator based on the Dietary Guidelines for Americans (DGA), and the HEI-2015 was the most recent version of the HEI. Multiple studies have discovered that the HEI score is related to health status, including physiological indicators (16), biochemical indicators (17), and disease risk (18, 19). The daily diet proposed by DGA is more adaptable in terms of food choices and is suited for a wider spectrum of people than the DASH diet and the Mediterranean diet (20). Furthermore, the adoption of the HEI-2015 also renders it possible to apply numerical values to represent how healthy a person’s daily diet is, allowing for a comparison of diet quality.

To the best of our knowledge, no studies have investigated the relationship between HEI-2015 and gout or HUA. Therefore, this study intends to employ the HEI-2015 to estimate the overall health impact of various dietary components, in addition to analyzing the risk of developing gout or HUA in terms of adhering to DGA.

## Materials and methods

### Study sample

The National Health and Nutrition Examination Survey (NHANES) is a large open database developed to better understand the nutrition and diet of the American population. The database utilized a unique and complex multistage probabilistic design, so that sample weights could be used to reflect the non-institutionalized population of the United States. All subjects received a dietary survey and examination by a professional at mobile exam centers (MECs). The examination includes medical, dental, and physiological measurements, as well as laboratory tests, which were supervised by trained medical personnel. In addition, a variety of modern equipment enables NHANES to collect reliable, high-quality data.

**FIGURE 1:**
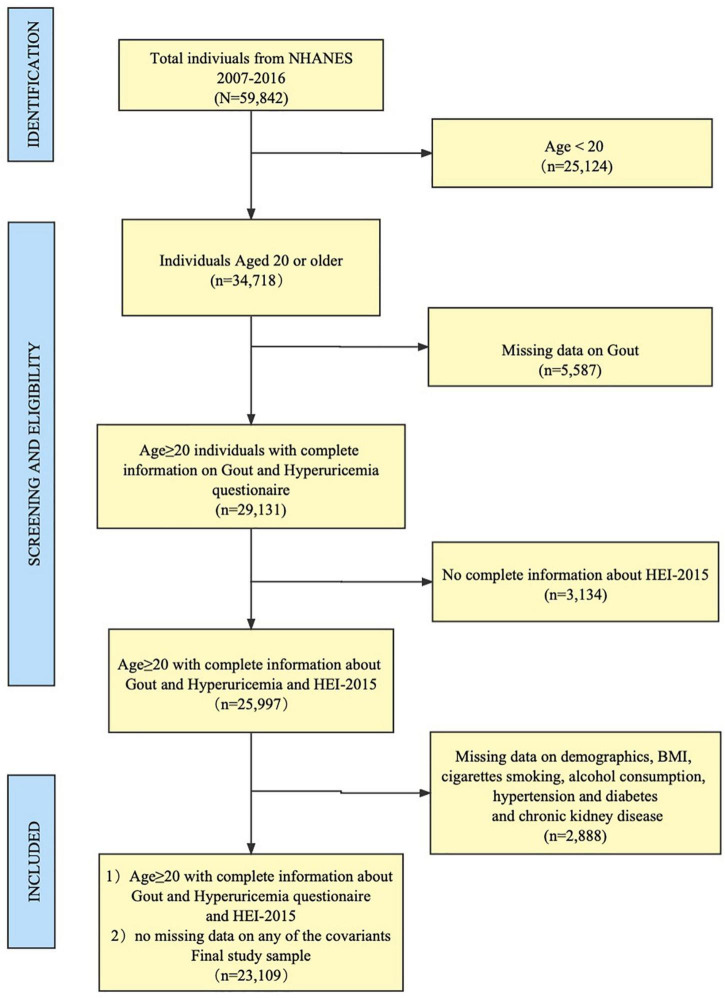
Flowchart of the study population.

We selected five consecutive survey cycles of NHANES (i.e., 2007–2008, 2009–2010, 2011–2012, 2013–2014, 2015–2016), and the overall sample size of adults (age ≥ 20) with no missing data for any variables is 23,109. The sample was weighted to represent a non-institutionalized adult population of 190 million Americans. Additional details of the study design, sampling, and exclusion criteria were illustrated in Figure 1. Only public data were used in the analysis, and ethical approval was not required for this study.

### Measurements and covariation assessment

#### Diet quality

HEI-2015 is utilized to assess the degree of adherence to DGA, which consists of 13 kinds of components (21). These 13 components were grouped into two forms, namely, the adequacy components (total vegetables, greens and beans, total fruits, whole fruits, whole grains, dairy, total protein foods, seafood and plant proteins, fatty acids) and the moderation components (sodium, refined grains, saturated fats, added sugars). Different maximum scores and weights were assigned to each component, and the overall 13 component scores (HEI-2015 scores) ranged from 0 to 100, with higher HEI scores reflecting better diet quality. Participants who had both two 24-h dietary recalls were included, and their dietary recall status was restricted to be reliable or had satisfied the minimum criteria for each day, which can reduce the bias of the HEI-2015. The 24-h dietary recall data were collected for 2 days, which were conducted by a trained interviewer face-to-face in the MEC on the first day and a follow-up interview 3-10 days later *via* phone. More details on dietary surveys and quality control can be found in the survey manual.^1^ The HEI-2015 total and component scores were calculated by the simple HEI scoring algorithm using publicly available SAS macros. For weighted Scott–Rao chi-square tests and weighted logistic regressions, quartiles were used to categorize the HEI-2015 score into four groups, recorded as Q_1_ (lowest diet quality, reference group), Q_2_, Q_3_, and Q_4_ (highest diet quality), respectively (22).

#### Gout

All subjects were asked “Has a doctor or other health professional ever told you that you had gout?” and then categorized into non-gout subjects (reference group) and gout subjects according to the answers (23).

#### Hyperuricemia (HUA)

The level of uric acid in blood, plasma, and urine was determined using a timed endpoint approach. Uric acid is oxidized by uricase to produce allatoin and hydrogen peroxide. The hydrogen peroxide reacts with 4-aminoantipyrine (4-AAP) and 3,5-dichloro-2-hydroxybenzene sulfonate (DCHBS) in a reaction catalyzed by peroxidase to produce a colored product. The system monitors the change in absorbance at 520 nm at a fixed time interval. More quality control details can be found in the documentation (24). The uric acid values were recorded in mg/dl and then converted to μmol/L by multiplying 59.48. A serum urate level of >7.0 mg/dl in men and >5.7 mg/dl in women was considered HUA (23).

### Covariates

#### Sex

Sex was classified as female (reference group) and male.

#### Age

Age was categorized into four groups, namely, 20–39 years group (reference group), 40–59 years group, 60–79 years group, and 80 years and above group (23).

#### Race

Race, according to NHANES classification, was categorized into non-Hispanic white (reference group), Mexican American group, non-Hispanic black, and other races (25).

#### Education

Education level was categorized as less than high school (reference group), high school graduate/GED, some college/AA degree, and college graduate or more (26).

#### Family income

Family income was categorized as ≤130% (reference group), >130–350%, and >350% by the ratio of family income to poverty (FPL) (27).

#### BMI status

Body mass index was calculated from measured height and weight as weight/height^2^ (kg/m^2^), then categorized into underweight (<18.5), normal (reference group, ≥18.5–24.9), overweight (≥25–29.9), and obese (≥30) (28).

#### Smoking status

Smoking behavior was measured by the “Smoking: Cigarette Use” questionnaire. The respondent was asked whether he/she had smoked at least 100 cigarettes in his/her life. If the respondent answered “no,” he/she was classified as a never smoker (reference group). If the respondent had smoked at least 100 cigarettes in his/her life and still smokes when he/she answers the questionnaire, he/she is classified as a current smoker. The respondent was classified as a former smoker who has smoked 100 cigarettes in his life and had quit smoking when answering the questionnaire (29).

#### Drinking status

Drinking behavior was measured in the “alcohol use” questionnaire. In the “alcohol use” questionnaire, each respondent was asked how often he/she had drunk alcoholic drinks in the past 12 months and the average drinks on those days that he/she drank alcoholic beverages. According to these questions, the average number of alcoholic drinks consumed per week in the past 12 months could be calculated. A “drink” was defined as a 12-ounce beer, a 5-ounce glass of wine, or one-and-half ounces of liquor. Then, it was categorized into four strata (0, <1, 1–8, and ≥8 drinks per week) and defined as none (reference group), light, moderate, and heavy alcohol consumption, respectively (30).

#### Hypertension

All subjects were asked, “Ever been told by a doctor or other health professional that you had hypertension?,” and then categorized into non-hypertension subjects (reference group) and hypertension subjects (31).

#### Chronic kidney disease

All subjects were asked, “Ever been told by a doctor or other health professional that you had weak or failing kidneys?,” and then categorized into non-CKD subjects (reference group) and chronic kidney disease (CKD) subjects (32).

#### Diabetes

During the NHANES home interviews, all subjects were asked, “Ever been told by a doctor or other health professional that you had diabetes or sugar diabetes?,” and then categorized into non-diabetic subjects (reference group) and diabetic subjects (33).

### Statistical analysis

Analyses were conducted according to the Centers for Disease Control and Prevention (CDC) guidelines for analysis of NHANES data. A full sample 2-year mobile examination centers (MEC) weight was used to calculate the US non-institutionalized population.

Linear regression models were adopted to analyze the trends in the prevalence of gout and HUA in the five consecutive cycles. The baseline features of different groups were examined using the Scott-Rao chi-square tests, and the association between the HEI-2015 score and the risk of gout/HUA was investigated using weighted logistic regression models.

The weighted quartile sum (WQS) (34–37) regression model was used to assess the effects of mixed exposure to thirteen dietary components of HEI-2015. The WQS regression model calculates a weighted regression index that represents the overall dietary health effect for all thirteen dietary components of HEI-2015. The WQS model functions as follows:


$$\text{g}\,\left(\mu \right)=\beta_{0}+\beta_{1}\,\left(\sum_{i=0}^{c}\omega_{i}\,q_{i}\right)+z^{\prime }\,\Phi$$

$$W\,Q\,S=\sum_{j=1}^{c}\varpi_{j}\,q_{j}$$

where β_0_ is the intercept; z′ and Φ represent the matrix of covariates and the coefficients of the covariates. *c* is the number of dietary components considered in the analysis, and 13 dietary components were included in the current analysis. The whole sum of weighted indices (ω_*i*_) is equal to 1, with the value of each component varying from 0 to 1$\left(\sum_{\text{i}=0}^{\text{c}}\omega_{\text{i}}{|}_{\text{b}}=1,0\geq \omega_{\text{i}}\geq 1\right).\beta_{1}$ is the regression coefficient of the WQS index. *q_j_* represents the quartiles of a dietary component score (= 0, 1, 2, or 3 for the first, second, third, or fourth quartile, respectively). *g*(μ) is a logit link function, when the outcome of interest is binary (gout or not, HUA or not). The corresponding weight of each dietary component showed how much a specific dietary component contributed to the WQS index. The data were randomly split into two data sets (40% as training set and 60% as validation set).

The corresponding weight of each dietary component showed how much a specific dietary component contributed to the WQS index. The data were randomly split into two data sets (40% as the training set and 60% as the validation set).

For all measures, we calculated 95% confidence intervals (CIs). The receiver operating characteristic curve (ROC) was used to validate the degree of WQS model fit.

All statistical tests were two-sided, and significance was considered at *P* < 0.05. WQS and ROC were performed with the R (version 4.1.0). RCS was implemented with the R package “rms” (version 6.3-0). WQS was implemented with the R package “gWQS” (version 3.0.4). Additional statistical analyses were performed using the SPSS statistical package (version 23.0; SPSS Inc., Chicago, IL, United States).

## Results

### Population characteristics between groups

Over the period of a total of five cycles from 2007 to 2016, the prevalence of gout (3.9% in 2007–2008 to 3.8% in 2015–2016, *P* = 0.519) and HUA (21.6% in 2007–2008 to 20.4% in 2015–2016, *P* = 0.161) among United States adults remained steady (Table 1).

**TABLE 1:** The prevalence of gout and HUA among United States adults from 2007 to 2016.

	2007–2008 (*n* = 4831)	2009–2010 (*n* = 5006)	2011–2012 (*n* = 4150)	2013–2014 (*n* = 4620)	2015–2016 (*n* = 4502)	*P*-value
No. gout	224	223	173	186	216	
The prevalence of gout (Weighted%)	3.9%	3.8%	3.8%	4.1%	3.8%	0.519
No.HUA	1,115	1,084	892	964	928	
The prevalence of HUA (Weighted%)	21.60%	21.10%	20.10%	19.70%	20.40%	0.116

HUA, Hyperuricemia.

The baseline characteristics of two groups (adults with/without gout) revealed that adults with gout were more likely to be male, over 60 years old, non-Hispanic black, have less than high school education, have low family income, obese, alcoholic, former smokers and suffer from CKD, hypertension, diabetes, HUA, and have lower HEI scores (Table 2).

**TABLE 2:** Characteristics among adults aged 20 years or older by gout.

Characteristics	Adults without gout (n, %)	Adults with gout (n, %)	*P*-value
**Sex no. (Weighted%)**			<0.001
Female	11,369 (51.77)	302 (30.6)	
Male	10,698 (48.23)	740 (69.4)	
**Age group no. (Weighted%)**			<0.001
20–39 year	7,296 (35.24)	54 (6.39)	
40–59 year	7,416 (37.74)	264 (34.17)	
60–79 year	5,966 (22.52)	557 (47.3)	
80+ year	1,389 (4.51)	167 (12.13)	
**Race no. (Weighted%)**			<0.001
Non-Hispanic white	9,615 (68.56)	560 (77.16)	
Mexican American	3,528 (8.62)	73 (3.04)	
Non-Hispanic black	4,367 (10.34)	265 (11.83)	
Other	4,557 (12.48)	144 (7.97)	
**Education no. (Weighted%)**			<0.001
<High school	5,417 (16.12)	269 (17.13)	
High school/GED	4,997 (22.09)	269 (24.27)	
College/AA degree	6,491 (31.86)	296 (32.01)	
College or above	5,162 (29.94)	208 (26.6)	
**Family income no. (Weighted%)**			<0.001
0∼130 FPL	6,496 (19.97)	309 (20.88)	
>130∼350 FPL	7,550 (33.22)	368 (32.41)	
>350 FPL	8,021 (46.82)	365 (46.71)	
**BMI no. (Weighted%)**			<0.001
Normal weight	5,861 (27.78)	143 (11.59)	
Underweight	330 (1.47)	6 (0.35)	
Overweight	7,352 (33.63)	307 (30.87)	
Obese	8,524 (37.11)	586 (57.19)	
**Drink level no. (Weighted%)**			<0.001
None	7,133 (26.02)	407 (34.53)	
Light	6,786 (30.65)	268 (25.86)	
Moderate	7,548 (40.63)	337 (36.39)	
Heavy	600 (2.69)	30 (3.22)	
**Smoke status no. (Weighted%)**			<0.001
Never smoker	12,282 (55.65)	429 (42.83)	
Former smoker	5,214 (24.4)	446 (42.16)	
Current smoker	4,571 (19.95)	167 (15.01)	
**CKD no. (Weighted%)**			<0.001
No	21,419 (97.69)	919 (90.71)	
Yes	648 (2.31)	123 (9.29)	
**Diabetes no. (Weighted%)**			
No	19,411 (91.1)	726 (75.52)	<0.001
Yes	2,656 (8.9)	316 (24.48)	
**Hypertension no. (Weighted%)**			<0.001
No	14,432 (69.22)	261 (30.13)	
Yes	7,635 (30.78)	781 (69.87)	
**HUA no. (Weighted%)**			<0.001
No	17,577 (80.47)	549 (53.71)	
Yes	4,490 (19.53)	493 (46.29)	
**HEI category no. (Weighted%)**			<0.001
Q_1_	5,492 (25.04)	233 (23.75)	
Q_2_	5,491 (25.02)	259 (24.7)	
Q_3_	5,590 (24.92)	294 (27.06)	
Q_4_	5 494 (25.02)	256 (24.49)	

Values are survey-weighted percentages. FPL, family income to poverty; CKD, chronic kidney disease; HUA, Hyperuricemia; HEI, healthy eating index.

Adults with HUA were more likely to be female, over 60 years old, non-Hispanic black, have a middle level of education, originate from low-income families, obese, alcoholics, or former smokers. They were also more likely to have CKD, hypertension, and diabetes, as well as lower HEI scores (Supplementary Table 1).

### Higher healthy eating index score is associated with a lower risk of gout

After stepwise adjusting for covariates, a binary logistic regression model revealed that decreased gout risk was related to higher HEI-2015 scores (Table 3). In the model adjusted for age, sex, race/ethnicity, family income, and education, higher diet quality was linked to significantly decreased risks of gout [odds ratio (OR) 0.832 95% confidence intervals (CI): 0.830–0.834 for Q_4_ compared with Q_1_, *P* < 0.001]. Additional adjustments for BMI, smoking, and alcohol consumption did not significantly weaken this connection (OR: 0.886, 95%CI: 0.884–0.888 for Q_4_ compared with Q_1_, *P* < 0.001). The connection between HEI-2015 and gout risk remained significant (OR: 0.878, 95% CI: 0.876–0.880 for Q_4_ compared with Q_1_, *P* < 0.001) after further adjustments for chronic disease characteristics, including hypertension, diabetes, CKD, and HUA.

**TABLE 3:** Relationship between HEI-2015 and gout among adults aged 20 years or older.

Variable	OR (95% CI)			*P*-value
	
	Model 1	Model 2	Model 3	
**Sex (reference, female)**				<0.001
Male	2.743 (2.739, 2.748)	2.643 (2.639, 2.648)	3.027 (3.022, 3.033)	
**Age group (reference, 20–39 year)**				<0.001
40–59 year	5.204 (5.188, 5.220)	4.522 (4.508, 4.537)	3.68 (3.668, 3.692)	
60–79 year	12.372 (12.334, 12.411)	9.993 (9.961, 10.025)	6.234 (6.213, 6.254)	
80+ year	17.066 (17.002, 17.129)	15.729 (15.669, 15.790)	8.465 (8.431, 8.499)	
**Race (reference, non-Hispanic white)**				<0.001
Mexican American	0.430 (0.428, 0.432)	0.398 (0.396, 0.400)	0.452 (0.450, 0.454)	
Non-Hispanic black	1.245 (1.242, 1.248)	1.231 (1.228, 1.234)	1.022 (1.019, 1.024)	
Other	0.756 (0.753, 0.758)	0.828 (0.826, 0.830)	0.809 (0.807, 0.812)	
**Education (reference, <high school)**				<0.001
High school/GED	1.046 (1.044, 1.049)	1.056 (1.054, 1.059)	1.098 (1.095, 1.101)	
College/AA degree	1.122 (1.120, 1.125)	1.113 (1.110, 1.115)	1.121 (1.118, 1.124)	
College or above	0.932 (0.929, 0.934)	1.061 (1.059, 1.064)	1.152 (1.148, 1.155)	
**Family income (reference,0∼130% FPL)**				<0.001
>130∼350% FPL	0.703 (0.702, 0.705)	0.676 (0.674, 0.677)	0.722 (0.721, 0.724)	
>350% FPL	0.700 (0.699, 0.702)	0.681 (0.679, 0.682)	0.774 (0.772, 0.776)	
**HEI category (reference, Q_1_)**				<0.001
Q_2_	0.910 (0.908, 0.912)	0.903 (0.901, 0.905)	0.888 (0.886, 0.890)	
Q_3_	0.974 (0.972, 0.976)	1.01 (1.007, 1.012)	0.991 (0.989, 0.993)	
Q_4_	0.832 (0.830, 0.834)	0.886 (0.884, 0.888)	0.878 (0.876, 0.880)	
**BMI (reference, normal weight)**				<0.001
Underweight		0.619 (0.611, 0.627)	0.689 (0.680, 0.698)	
Overweight		1.678 (1.674, 1.683)	1.361 (1.358, 1.365)	
Obese		3.062 (3.054, 3.069)	1.907 (1.902, 1.912)	
**Drink level (reference, none)**				<0.001
Light		0.822 (0.820, 0.823)	0.871 (0.869, 0.872)	
Moderate		0.878 (0.876, 0.879)	0.913 (0.912, 0.915)	
Heavy		1.314 (1.308, 1.320)	1.203 (1.197, 1.209)	
**Smoke status (reference, never)**				<0.001
Former		1.335 (1.332, 1.337)	1.266 (1.264, 1.268)	
Current		1.019 (1.017, 1.022)	1.05 (1.047, 1.052)	
**CKD (reference, no)**				<0.001
Yes			2.051 (2.045, 2.057)	
**Diabetes (reference, no)**				<0.001
Yes			1.33 (1.327, 1.332)	
**Hypertension (reference, no)**				<0.001
Yes			2.304 (2.300, 2.308)	
**HUA (reference, no)**				<0.001
Yes			2.484 (2.480, 2.488)	

FPL, family income to poverty; CI, confidence interval; OR, odds ratio; CKD, chronic kidney disease; Model 1, adjusted for demographic characteristics (sex, age group, race, education, family income); Model 2, adjusted for demographic characteristics (sex, age group, race, education, family income); BMI, smoking, and drinking status; Model 3, adjusted for demographic characteristics (sex, age group, race, education, family income); BMI, smoking, drinking status, hypertension, CKD, diabetes, and hyperuricemia.

By applying the same analytical technique, we also discovered that higher HEI-2015 scores were related to a decreased risk of HUA (Q4 OR: 0.978, 95% CI: 0.976–0.979 for Q_4_ compared with Q_1_, *P* < 0.001, see Supplementary Table 2).

**FIGURE 2:**
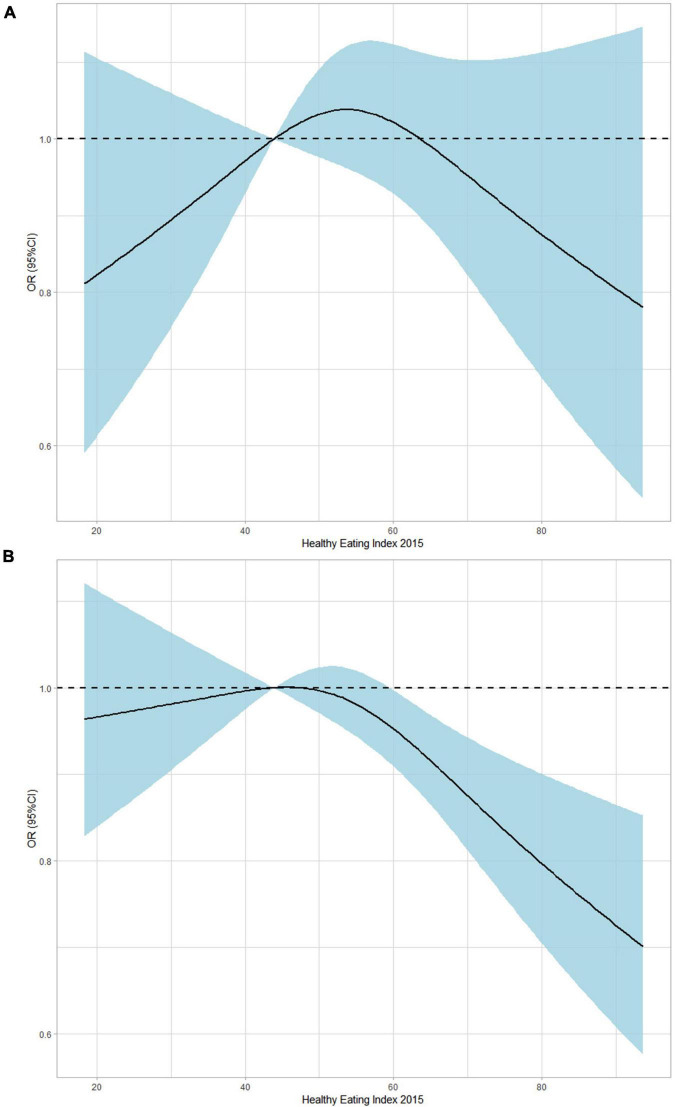
Dose-response association between HEI (it continues) and gout/HUA using restricted cubic splines (RCS). The models of gout **(A)** were adjusted for sex, age group, race, education, family income, BMI, smoking, drinking status, hypertension, CKD, diabetes, and hyperuricemia. The models of HUA **(B)** were adjusted for sex, age group, race, education, family income, BMI, smoking, drinking status, hypertension, CKD, and diabetes.

Figure 2 demonstrated the dose-response relationship between gout/HUA and HEI-2015 (as a continuous variable) by applying the restricted cubic splines (RCS) approach. Higher HEI-2015 scores did not demonstrate a protective effect of gout (OR: 0.988, 95% CI: 0.973–1.003, *P* = 0.1223) and instead showed a declining trend in OR (Figure 2A). Additionally, higher HEI-2015 scores revealed a declining OR trend (OR: 0.992, 95% CI: 0.985–0.999, *P* = 0.0386) and the preventive effects of HUA (Figure 2B).

### Mixed effects of 13 dietary components on gout

The WQS regression was used to evaluate the health contributions of various dietary components to explore the health implications of dietary components in the total diet.

The WQS indices were statistically associated with gout. After adjusting for demographic variables, including age, sex, race, household income, and education level, the WQS index was significantly associated with progressively lower odds of gout (OR: 0.955, 95% CI, 0.930–0.982, *P* = 0.0009). The WQS index was significantly correlated with decreased risks of gout after further adjustments for BMI, smoking, and alcohol use (OR: 0.957, 95% CI, 0.933–0.983, *P* = 0.0011). These correlations were not significantly weakened by further adjustments for chronic disease characteristics (hypertension, diabetes, CKD, HUA) (OR: 0.963, 95% CI: 0.937–0.990, *P* = 0.0067).

Additionally, we discovered that the WQS indices significantly correlated with HUA (Supplementary Table 3). The WQS index was significantly linked with progressively decreased odds of HUA after multiple adjustments (OR: 0.934, 95% CI: 0.919–0.950, *P* < 0.0001).

**FIGURE 3:**
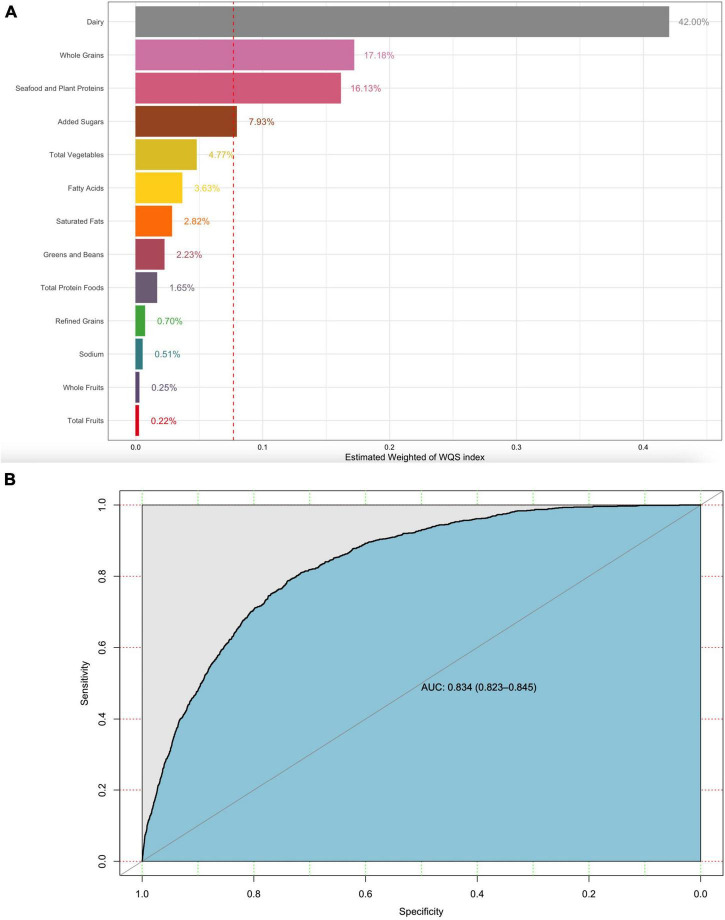
WQS model regression index weights for gout **(A)** and the AUCs of the WQS models **(B)**. Models were adjusted for sex, age group, race, education, family income, BMI, smoking, drinking status, hypertension, CKD, diabetes, and hyperuricemia.

**FIGURE 4:**
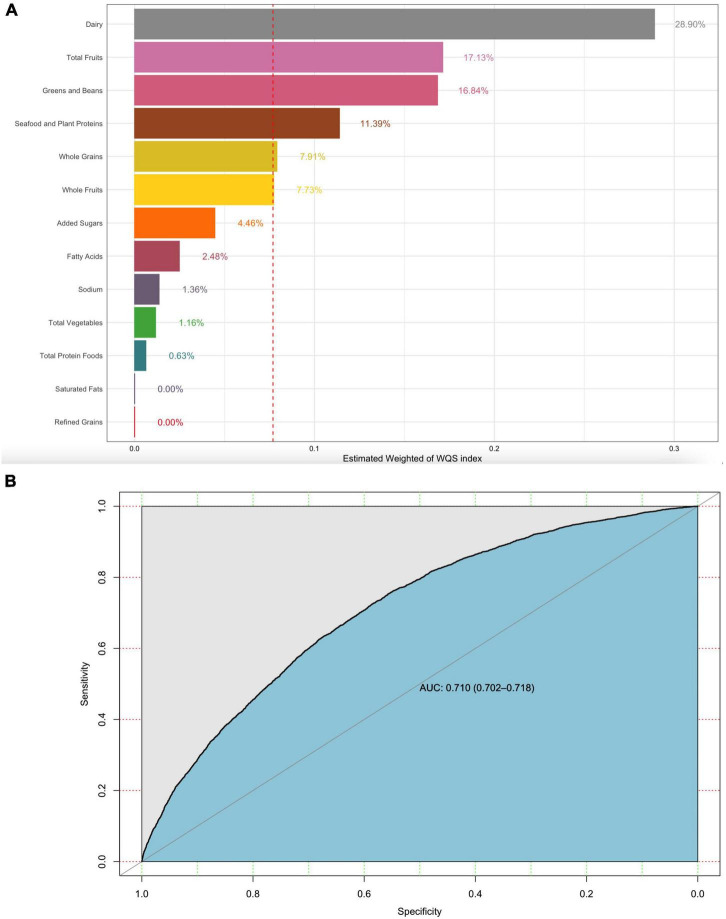
WQS model regression index weights for HUA **(A)** and the AUCs of the WQS models **(B)**. Models were adjusted for sex, age group, race, education, family income, BMI, smoking, drinking status, hypertension, CKD, and diabetes.

The weight of each dietary component in the WQS regression model represented the contributions of the overall dietary health effects. Dairy, whole grains, and plant proteins were the highest weighted dietary components in the model of gout (42, 17.18, and 16.13%, respectively). Added sugar as a moderation component was also the highest weighted dietary component in the model of gout (7.93%) (Figure 3A). Similarly, dairy, total fruits, greens, beans, and plant proteins were the highest weighted dietary components in the model of HUA (Figure 4A) (28.9, 17.13, 16.84, and 11.39%, respectively). The evaluation of the WQS models showed that the area under the ROC curves (AUCs) was 0.834 in gout (Figure 3B) and 0.710 in HUA (Figure 4B).

## Discussion

In agreement with the American College of Rheumatology report (23), this study discovered that the prevalence of both gout and HUA remained steady from 2007 to 2016. Our stepwise logistic regression models demonstrated that higher HEI-2015 scores were independently related to a decreased risk of gout/HUA. The findings supported the concept of HEI-2015’s recommendations for a healthy diet (20, 21), which noted that a high-quality diet improved the quality of life.

Patients with HUA who followed a Mediterranean diet showed a decrease in blood uric acid concentration of 119 μmol/L over 6 months in a randomized controlled study (*P* for within-group comparison < 0.001)[9]. The quality of the Mediterranean diet, however, was not measured in this study. After quantifying the quality of the Mediterranean diet, MD Kontogianni et al. found a 70% reduction in the risk of HUA in those scoring in the Q_4_ of the Mediterranean diet compared with those scoring in the Q_1_ (OR: 0.30, 95%CI: 0.11–0.82) (38). According to a prospective cohort study of adult men conducted by Rai et al., higher DASH dietary scores were linked to a decreased incidence of gout (OR: 0.68, 95% CI: 0.57–0.80, *P*< 0.001, Q_5_ vs. Q_1_) (8). However, certain studies, such as those on the Mediterranean diet (39, 40) and the DASH diet (41, 42), estimated dietary quality scores based on the frequency of meals. In our study, we determined the consumption of each dietary component and then estimated the HEI-2015 score in the form of nutritional density (43). The HEI-2015 has 13 dietary components, compared with the 11 components of the Mediterranean diet score and the 8 components of the DASH diet, which improves the validity of our findings.

The HEI-2015 score calculation was based on the DGA, which included daily dietary recommendations for the US population and sought to improve dietary quality (20, 44). We concentrated on the contributions of different components to identify the component that contributed the most to offering better dietary guidance to individuals with gout/HUA and at risk of gout/HUA. The results indicated that added sugars, dairy products, whole grains, seafood, and plant proteins contributed the most to lowering the risk of gout in the HEI-2015, implying that increasing dietary intake of dairy products, whole grains, vegetables, and legumes, as well as lowering added sugar intake within the recommended range, may decrease the risk of gout. Additionally, the diet’s ability to protect against HUA was most strongly influenced by dairy products, total fruits, greens and beans, seafood and vegetable protein, whole grains, and whole fruits. It was shown that consuming more dairy products, whole grains, fruits, vegetables, and legumes while consuming added sugars less frequently and within the recommended range can lower the risk of HUA.

Dairy products provide a lot of high-quality protein, and it has been shown that casein and whey protein from milk can lower blood uric acid levels in healthy people (45). Dairy products are also low in purines and contribute less to the purine load associated with other high-quality protein sources, such as meat and seafood (10). Proteins and lipids from dairy products were also found to inhibit inflammatory responses associated with monosodium urate monohydrate (MSU) crystals *in vivo*/*in vitro* experiments (46). A study based on NHANES data revealed that a higher ratio of refined grains to whole grains was associated with a greater risk of CKD, and a high intake of whole grains was associated with low serum uric acid levels (47). Whole grains are a rich source of fiber, minerals, vitamins, phenolic compounds, phytoestrogens, and related antioxidants (48). These ingredients are beneficial for disease prevention and management (49–52). The association between fruit and uric acid/gout is disputed, since fructose metabolism generated urates, and fresh fruit is high in fructose (53, 54). Some studies have also found that fruit intake reduces the risk of gout because fruits contribute to urine alkalization and promote uric acid excretion (55). Fruits are rich in various nutrients, such as vitamin C (56, 57), potassium (58, 59), fiber (60), epicatechin, and flavonols (61, 62), which may alter the effects of fructose and urate. Studies on added sugars and gout have also indicated that reducing added sugar intake helps to decrease uric acid and prevent gout.

According to the Dietary Guidelines for Americans 2020–2025 (20), adults in the United States should consume 8 ounces of seafood per week; however, in our study, 79% of participants did not reach this recommended standard. However, we found that the seafood and plant protein components have a healthy contribution to gout/HUA. We assumed that the protective effects exhibited by the seafood and plant protein were due to seafood intake within reasonable limits (63) and the protective effects of plant protein (64, 65). The impact of low-dose seafood consumption and uric acid in healthy individuals required more exploration. Encouragement of greater consumption of dairy products, whole grains, low-fructose fruits, and high-quality protein from plant sources, particularly legumes, is crucial for preventing gout and decreasing blood uric acid.

We discovered additional risk variables for gout/HUA, including male gender, advanced age, non-Hispanic black race, middle education level, low income, obesity, alcohol intake, hypertension, and CKD. Due to estrogen’s stimulation of uric acid excretion, women have always had low rates of HUA and gout (3). Poor education and income levels are risk factors for gout because they are directly associated with socioeconomic position, which affects access to healthcare and treatment compliance (66). Drinking promotes uric acid metabolism, raises blood uric acid levels, and increases the risk of gout/HUA, which was consistent with our results (67). Aging, obesity, hypertension, and CKD are all common gout risk factors, raising the incidence of gout by affecting uric acid metabolism or excretion (3). Interestingly, we found that smoking was a protective factor for HUA, as smoking may lower blood uric acid levels by metabolically interacting with superoxide (68). Another rationale is that since there was only one blood uric acid test implemented in this study, it cannot accurately represent long-term uric acid levels. However, the damage of long-term smoking to overall health also increases the risk of gout (69).

Diabetes was found to be a protective factor for HUA in this investigation, according to the logistic regression results of the HUA model. Diabetes, as a complication of HUA, has a very close relationship with HUA. Our study discovered that the added sugar scores in diabetes patients were substantially higher than those in non-diabetic subjects, indicating a decreased intake of added sugar in diabetic people. Research indicated that added sugar intake may raise serum uric acid levels (70, 71) and raise the risk of developing gout (72). In the liver, the metabolism of fructose, a major source of added sugar in the daily diet, stimulates adenosine monophosphate deaminase which promotes the degradation of adenosine monophosphate to inosine monophosphate. This process could induce insulin resistance (73–75) and promote the production of uric acid in the liver (43), thereby increasing the risk of HUA. Therefore, we assumed that diabetes showed as a protective factor for HUA in the results due to the significantly lower intake of added sugars in diabetic subjects than in non-diabetic subjects. Then, we proposed that restricting added sugar consumption might significantly lower the risk of HUA, including the diabetic population.

The article had several limitations due to the nature of the study. As with any observational study, our estimates were still subject to residual and unmeasured confounders, and no causal relationship can be inferred. Second, self-reported data about disease were subject to recall bias, and lack of adjustment for medication history for disease would affect results. Finally, only one serum uric acid test did not reflect the long-term uric acid level of the subject and thus caused possible erroneous results.

Nonetheless, our study also has some strengths. A major strength is the use of a large, nationally representative database. All dietary interviewers were required to complete professional courses and training and regularly reinforce training annually. Data from two 24-h dietary survey were used to reduce recall bias from the food frequency questionnaire. The HEI was developed and validated using HNANES dietary data, and dietary data were collected in the same manner as the HEI-2015 score. Effective and reasonable quantification of dietary quality is also a strength of this article. Second, the WQS model had been used in fewer nutrition-related studies. We used the WQS model to identify the highest contributing dietary components, and this study was a new application of the WQS model. Through this article, we hope to make the public aware of the benefits of a healthier daily diet for gout and HUA.

## Conclusion

We found that people with the highest HEI-2015 score had a reduced risk of gout and HUA by 12.2 and 2.2% compared to people with the lowest HEI-2015 score. Dairy products, whole grains, fruits, and plant protein-related foods contributed the most to the health effects of the daily diet represented by HEI-2015. Therefore, people at high risk of gout or gout sufferers should pay more attention to maintaining a healthy diet and follow the DGA, especially increasing the intake of dairy products, whole grains, fruits, and legumes and reducing the intake of added sugars.

## Data availability statement

The original contributions presented in this study are included in the article/Supplementary material, further inquiries can be directed to the corresponding author/s.

## Author contributions

All authors listed have made a substantial, direct, and intellectual contribution to the work, and approved it for publication.

## Footnotes

1
https://wwwn.cdc.gov/nchs/nhanes/continuousnhanes/manuals.aspx?BeginYear=2015
