# Supplementary material for: Higher HEI-2015 scores are associated with lower risk of gout and hyperuricemia: Results from the national health and nutrition examination survey 2007–2016
Source: Front Nutr. 2022 Aug 3;9:921550. doi: 10.3389/fnut.2022.921550 (PMC9381708; doi:10.3389/fnut.2022.921550)
Supplement: Supplementary file 1 [file Data_Sheet_1.PDF]

Supplementary Material

Supplemental Table S1 Characteristics among adults aged 20 years or older by HUA

Characteristics	Adults Without HUA(n,%)	Adults With HUA(n,%)	*P-Value*
**Sex No. (Weighted%)**			<0.001
Female	9080(50.85)	2591(51.31)	
Male	9046(49.15)	2392(48.69)	
**Age Group No. (Weighted%)**			<0.001
20-39 y	6150(35.69)	1200(28.05)	
40-59 y	6198(38.44)	1482(34.33)	
60-79 y	4719(21.78)	1804(30.08)	
80+ y	1059(4.1)	497(7.53)	
**Race No. (Weighted%)**			<0.001
Non-Hispanic White	7878(68.3)	2297(71.23)	
Mexican American	3008(8.95)	593(6.28)	
Non-Hispanic Black	3404(9.92)	1228(12.22)	
Other	3836(12.83)	865(10.28)	
**Education No. (Weighted%)**			<0.001
< High School	4463(16.16)	1223(16.13)	
High school /GED	4045(21.59)	1221(24.41)	

College/AA degree	5232(31.07)	1555(34.93)	
College or above	4386(31.17)	984(24.53)	
**Family Income No. (Weighted%)**			<0.001
0~130% FPL	5330(19.95)	1475(20.18)	
>130%~350% FPL	6127(32.5)	1791(35.84)	
>350% FPL	6669(47.55)	1717(43.98)	
**BMI No. (Weighted%)**			<0.001
Normal Weight	5381(31.03)	623(12.21)	
Underweight	312(1.72)	24(0.32)	
Overweight	6260(34.77)	1399(28.71)	
Obese	6173(32.49)	2937(58.76)	
**Drink Level No. (Weighted%)**			<0.001
None	5753(25.64)	1787(29.09)	
Light	5670(31.02)	1384(28.35)	
Moderate	6235(40.85)	1650(39)	
Heavy	468(2.5)	162(3.56)	
**Smoke Status No.(Weighted%)**			<0.001
Never Smoker	10079(55.62)	2632(53.34)	
Former Smoker	4200(23.99)	1460(29.34)	
Current Smoker	3847(20.39)	891(17.32)	
**CKD No. (Weighted%)**			<0.001
No	17671(98.1)	4667(94.8)	
Yes	455(1.9)	316(5.2)	
**Diabetes No. (Weighted%)**			<0.001

	No	16073(91.67)	4064(85.98)	
	Yes	2053(8.33)	919(14.02)	
**Hypertension No. (Weighted%)**				<0.001
	No	12462(72.24)	2231(50.16)	
	Yes	5664(27.76)	2752(49.84)	
**HEI Category No. (Weighted%)**				<0.001
	Q_1	4482(24.92)	1243(25.26)	
	Q_2	4446(24.7)	1304(26.19)	
	Q_3	4619(25.06)	1265(24.77)	
	Q_4	4579(25.32)	1171(23.78)	

Values are survey-weighted percentages. HUA=Hyperuricemia, FPL = family income to poverty. CKD=chronic kidney disease. HEI = healthy eating index.

Supplemental Table S2 Relationship between HEI and HUA among Adults aged 20 years or older.

Variable	*OR(95%CI)*			*P-Value*
	Model 1	Model 2	Model 3	
**Sex (reference, Female)**				<0.001
Male	1.014(1.014,1.015)	0.943(0.942,0.943)	0.944(0.944,0.945)	
**Age group (reference, 20-39 y)**				<0.001
40-59 y	1.157(1.156,1.158)	1.007(1.006,1.008)	0.879(0.878,0.88)	
60- 79 y	1.781(1.779,1.783)	1.536(1.534,1.538)	1.149(1.148,1.15)	
80+ y	2.305(2.301,2.308)	2.514(2.509,2.518)	1.732(1.729,1.735)	
**Race (reference, Non-Hispanic White)**				<0.001
Mexican American	0.729(0.728,0.73)	0.613(0.612,0.614)	0.639（0.638,0.64）	
Non-Hispanic Black	1.21(1.209,1.211)	1.102(1.1,1.103)	1.041(1.039,1.042）	
Other	0.849(0.848,0.85)	0.925(0.923,0.926)	0.921(0.92,0.922）	
**Education (reference, < High School)**				<0.001
High school /GED	1.097(1.096,1.098)	1.067(1.066,1.069)	1.088(1.086,1.089）	
College/AA degree	1.146(1.145,1.148)	1.092(1.09,1.093)	1.099(1.098,1.101）	
College or above	0.83(0.829,0.831)	0.878(0.877,0.879	0.905(0.903,0.906）	
**Family Income (reference, 0~130% FPL)**				<0.001
>130%~350% FPL	1.024(1.023,1.025)	0.991(0.99,0.992)	1.022(1.021,1.023）	
>350% FPL	0.931(0.93,0.932)	0.9(0.899,0.901)	0.939(0.938,0.94）	
**HEI Category (reference, Q_1)**				<0.001
Q_2	1.014(1.013,1.015)	1.012(1.011,1.013)	1.007(1.005,1.008）	
Q_3	0.939(0.938,0.94)	0.969(0.968,0.97)	0.973(0.972,0.974）	

Q_4	0.888(0.887,0.889)	0.968(0.967,0.97)	0.978(0.976,0.979）	
**BMI (reference, Normal Weight)**				<0.001
Underweight		0.483(0.481,0.486)	0.477(0.475,0.48）	
Overweight		2.079(2.076,2.081)	1.973(1.97,1.975）	
Obese		4.679(4.674,4.684)	4.145(4.14,4.149）	
**Drink Level (reference, None)**				<0.001
Light		0.907(0.906,0.908)	0.933(0.932,0.934）	
Moderate		1.186(1.185,1.187)	1.23(1.229,1.231）	
Heavy		1.875(1.871,1.88)	1.883(1.879,1.888）	
**Smoke Status (reference, Never)**				<0.001
Former		1.036(1.036,1.037)	1.007(1.006,1.008）	
Current		0.904(0.903,0.905)	0.884(0.883,0.885）	
**CKD (reference, No)**				<0.001
Yes			2.16(2.155,2.164）	
**Diabetes (reference, No)**				<0.001
Yes			0.961(0.96,0.962）	
**Hypertension (reference, No)**				<0.001
Yes			1.822(1.82,1.823）	

FPL = family income to poverty; CI = confidence interval; OR = odds ratio. CKD=chronic kidney disease.

Model 1 = adjusted for demographics characteristics (sex, age group, race, education, family income);

Model 2 = adjusted for demographics characteristics (sex, age group, race, education, family income), BMI, smoking, and drinking status;

Model 3= adjusted for demographics characteristics (sex, age group, race, education, family income), BMI, smoking, drinking status, hypertension, CKD, and diabetes.

Supplemental Table S3 Relationship between WQS regression index and gout and hypernricemia among Adults aged 20 years or older

Outcome		OR	95%CI	*P* value
**Gout**				
	Model 1	0.955	(0.930 ,0.982)	0.0009
	Model 2	0.957	(0.933 ,0.983)	0.0011
	Model 3	0.963	(0.937 ,0.990)	0.0067
**Hypernricemia**				
	Model 1	0.927	(0.911 ,0.943)	<0.0001
	Model 2	0.933	(0.918 ,0.949)	<0.0001
	Model 3	0.934	(0.919 ,0.950)	<0.0001

Model of gout:Model 1 = adjusted for demographics characteristics (sex, age group, race, education, family income);

Model 2 = adjusted for demographics characteristics (sex, age group, race, education, family income), BMI, smoking, and drinking status;

Model 3= adjusted for demographics characteristics (sex, age group, race, education, family income), BMI, smoking, drinking status, hypertension, CKD, diabetes, hyperuricemia.

Model of Hypernricemia:Model 1 = adjusted for demographics characteristics (sex, age group, race, education, family income);

Model 2 = adjusted for demographics characteristics (sex, age group, race, education, family income), BMI, smoking, and drinking status;

Model 3= adjusted for demographics characteristics (sex, age group, race, education, family income), BMI, smoking, drinking status, hypertension, CKD, and diabetes.
